# Sperm-Associated Mercury Species Levels Determine Its Impairment: Evidence from Paired Analyses of Whole Semen and Seminal Plasma

**DOI:** 10.1007/s12011-026-05181-8

**Published:** 2026-06-16

**Authors:** Mengwei Yuan, Martin Tsz-Ki Tsui, Murong Xu, Chi Chiu Wang, David Yiu Leung Chan

**Affiliations:** 1 School of Life Sciences, The Chinese University of Hong Kong, Shatin, N.T., Hong Kong SAR China; 2 Department of Earth and Environmental Sciences, The Chinese University of Hong Kong, Shatin, N.T., Hong Kong SAR China; 3 State Key Laboratory of Marine Environmental Health, City University of Hong Kong, Kowloon Tong, Kowloon, Hong Kong SAR China; 4 Assisted Reproductive Technology Unit, Department of Obstetrics and Gynaecology, The Chinese University of Hong Kong, Hong Kong SAR, China; 5 Department of Obstetrics and Gynaecology, Prince of Wales Hospital, Faculty of Medicine, CUHK, 30-32 Ngan Shing Street, Hong Kong, China

**Keywords:** Mercury species, Semen quality, Exposure assessment, Male reproductive health, Environment

## Abstract

There have been conflicting results regarding the relationship between mercury (Hg) and semen quality among previous studies, partly because most studies rely on indirect reproductive biomarkers (e.g., whole blood, hair) and no study tested Hg species to understand their specific associations with semen quality. In this study, we collected paired whole semen and seminal plasma samples from 250 male participants aged 26–63 years old in Hong Kong. Total mercury (THg) and methylmercury (MeHg) levels were quantified in both matrices, and inorganic mercury (iHg) level was calculated based on the difference between THg and MeHg. We applied logistic and linear regression to examine associations between Hg levels and semen quality parameters, including normal morphology, total motility, and the number of abnormal semen parameters. To the best of our knowledge, this was the first study reporting Hg species concentrations (THg, MeHg, iHg) in human whole semen and seminal plasma samples. We found that the majority of Hg in whole semen and seminal plasma were iHg (65–70%). Interestingly, increasing MeHg levels in whole semen would accompany with improving semen quality. In contrast, we showed clearly that both iHg and MeHg, when normalized to total sperm count, would be positively associated with sperm morphology and motility impairment. This study clarified a longstanding problem how Hg content is correlated with semen quality, and the findings demonstrated that iHg, not MeHg, is the dominant form while Hg associated with sperm cells is the key determining factor of its toxicity to the male reproductive system.

**Supplementary Information:**

The online version contains supplementary material available at 10.1007/s12011-026-05181-8.

## Introduction

Infertility has become a serious global health concern, affecting 8–12% of reproductive age couples. Among many factors, male factor contributes to 20–30% of the overall infertility [3], but the causes of male subfertility can be numerous which remain poorly understood [59]. Among potential risk factors, environmental and occupational exposure to toxic chemicals has recently received increasing attention [71].

Mercury (Hg), a naturally occurring element that can be transported globally via long-range atmospheric transport and landed via dry and wet deposition [48]. Once landed, inorganic Hg [*iHg*; e.g., Hg(II)] can be converted by a suite of anaerobic microbes in reducing environments (e.g., surface sediments) into highly toxic methylmercury (*MeHg*) [62]. In fact, MeHg can extensively bioaccumulate at the base of aquatic food webs and increase its concentrations along the food chain, leading to very high levels in the top predators in the aquatic environment [39]. In the general population, MeHg exposure is largely driven by fish and seafood consumption, whereas elemental Hg (*Hg*^*0*^) exposure is commonly associated with dental amalgams [7, 30]. Occupational Hg exposure, in contrast, is mainly relevant to specific populations, such as artisanal gold miners and dental professionals [8, 32].

Experimental studies using mammalian animal models have consistently shown that exposure to Hg compounds across a range of routes and doses can impair male reproductive functions, including disrupted spermatogenesis, reduced sperm motility and histopathological damage to the seminiferous epithelium [1, 5, 28, 40, 53]. Within the testis, the blood-testis barrier (BTB), formed by desmosomes, gap junctions, tight junctions, and basal ectoplasmic specialization, create a protective essential microenvironment for spermatogenesis [47, 49, 75]. Animal studies have previously identified that exposure to Hg disrupts the BTB by inducing oxidative stress, cytoskeletal disorganization, and junctional protein loss, leading to increased barrier permeability, impaired sperm quality, and reduced fertility [38, 43–45, 68]. However, translating these findings to human populations remains limited and challenging [6].

A number of previous clinical studies attempted to relate Hg measurements in human tissue samples to semen parameters, but conflicting results have been demonstrated (*see* a summary in Table 1). For example, an early study in Hong Kong observed a significant correlation between male subfertility and total Hg level (i.e., addition of iHg and MeHg; *THg*) in the hair samples of men aged 25 to 75, and subfertile men had around 40% higher THg than fertile men of similar age [24]. Similarly, Choy et al., [18] reported a positive association between semen THg levels and aberrations in sperm morphology and sperm motility in males in Hong Kong. Ai et al., [4] showed that participants from the Reproductive Medicine Center in Taiwan had higher abnormal sperm morphology, potentially associated with predatory fish intake and high blood THg level. In a more recent study, Henriques et al., [35] found that Portuguese men with higher total Hg levels in scalp hair exhibited significant correlations with sperm principal piece defects and elevated teratozoospermia index, indicating that Hg bioaccumulation may contribute to abnormal sperm morphology and impaired male fertility.

**Table 1:** Summary of previous studies on mercury (Hg) exposure biomarkers and semen quality outcomes

Study, Location	Population	*N*	Samples	Hg species	Main findings
Dickman et al., [24], Hong Kong	Fertile and subfertile men	166	Hair	THg	Higher hair Hg levels were associated with an increased risk of male subfertility in Hong Kong.
Choy et al., [18], Hong Kong	Male partners of infertile couples	111	Seminal fluid, whole blood	THg	Seminal fluid Hg was significantly associated with poorer sperm morphology and abnormal sperm motion patterns.
Meeker et al., [54], United States	Fertile men and men with fertility problems	219	Whole blood	THg	No significant relationship between blood Hg levels and semen quality parameters.
Mendiola et al., [55], Spain	Fertile men and men with fertility problems	61	Seminal plasma, whole blood, blood plasma	THg	No significant relationship between Hg levels in any matrix and semen quality parameters.
Kim et al., [41], United States	Male partners of couples undergoing IVF	30	Seminal plasma	THg	Positive associations for seminal plasma Hg and embryo quality, implantation, pregnancy, and live birth.
Mínguez-Alarcón et al., [56], United States	Male partners of couples seeking infertility treatment	129	Hair	THg	Higher Hg level was associated with sperm concentration, count and motility, but associations attenuated after adjustment for fish intake.
Ai et al., [4], Taiwan	Participants from a reproductive medical center	84	Seminal plasma, whole blood	THg	Participants with predatory fish intake and high blood Hg level had lower sperm with a normal morphology.
Henriques et al., [35], Portugal	Men attending Urology consultations	30	Hair	THg	Positive correlations between hair THg and sperm principal piece defects and teratozoospermia index.

In contrast, Meeker et al., [54] found no clear relationship between blood THg levels and semen quality among men from infertility clinics in the United States. Similarly, Mendiola et al., [55] analyzed the associations between seminal and hormonal parameters and the concentration of THg in Spanish men attending infertility clinics, observing no significant associations between THg levels in any matrix (whole blood, blood plasma, seminal plasma) and semen quality parameters. Interestingly, a few studies even showed positive correlations between THg levels in human biological samples and semen parameters. Specifically, Mínguez-Alarcón et al., [56] reported a positive association between hair THg levels and sperm concentration, total sperm count, and progressive motility among men prior to adjusting for other factors. In addition, a study of 30 men in the United States using in vitro fertilization found positive associations between seminal plasma THg level and embryo quality, implantation, pregnancy, and live birth [41]. Thus, no clear conclusion can be drawn from these previous studies regarding the relationship between THg and semen quality.

Human semen is a composite fluid in which spermatozoa, generated in the testis and matured in the epididymis, are suspended in seminal plasma [66]. Seminal plasma, constituting over 90% of the ejaculate volume, is a distinct mixture of secretions from the seminal vesicles, prostate, and bulbourethral glands, rich in fructose, citric acid, prostaglandins, and antioxidant enzymes [72, 80]. Because seminal plasma and spermatozoa originate from different anatomical compartments, the Hg profiles (i.e., Hg forms and concentrations) may differ between seminal plasma (extracellular compartment) and whole semen (combined extracellular and intracellular compartments), with potential implications for Hg exposure interpretation.

In this work, we postulated that distinguishing the specific forms of Hg, i.e., iHg vs. MeHg, would add new insights into resolving the ambiguous roles of Hg on semen quality. Indeed, the studies we reviewed only measured THg in the patient samples (Table 1), but iHg and MeHg can have different absorption, distribution, metabolism and in-vivo biological effects [9, 21]. It is known that MeHg has considerably higher bioavailability than iHg, MeHg can be absorbed nearly completely in the gastrointestinal tract and flows into the blood. Subsequently, MeHg rapidly distributes to various tissues, and through forming thiol conjugates (e.g., MeHg-L-cysteine) that structurally resemble amino acid methionine, MeHg gains a unique ability to across membranes, like the blood-brain barrier and placenta [36]. Intracellularly, MeHg can deplete antioxidant defenses, trigger oxidative stress and mitochondrial dysfunction, and eventually promote cellular injury and death [79].

In contrast, iHg (e.g., Hg^2+^) is inefficiently absorbed in the gastrointestinal tract with the absorption rate at or below 15% [79]. After entering circulation, iHg tends to bind strongly to thiol groups, and mainly accumulate in the kidney and then is eliminated primarily via urine, leading to a major distributional difference between iHg and MeHg [57]. Therefore, by monitoring just the THg in various human tissues may obscure specific kinetics of Hg forms and their exposure history, impeding our understanding on the Hg health risk and their associations with various health parameters.

In this study, we measured both THg and MeHg in paired whole semen and seminal plasma samples, and derive iHg through calculating the differences between THg and MeHg. Specifically, we examined iHg and MeHg in paired whole semen and seminal plasma samples from 250 men undergoing routine semen analysis in Hong Kong. To the best of our knowledge, this was the first study reporting Hg species concentrations (THg, MeHg, iHg) in human whole semen and seminal plasma samples. Given the high seafood consumption in Hong Kong (i.e., the second highest fish consumption per capita in Asia, only behind the oceanic island country, Maldives, and ranked the seventh highest in the world), this population provided an ideal model for studying Hg exposure through high consumption of both local and imported seafood [17]. We compared matrix-specific Hg forms and evaluated their associations with semen quality parameters to better characterize how Hg species and their specific exposures relate to male reproductive health.

## Materials and Methods

### Study Population and Sample Collection

This study was ethically approved by the Joint Chinese University of Hong Kong - New Territories East Cluster Clinical Research Ethics Committee (CREC No. 2016.499). A total of 250 male subjects (26–63 years old) of Hong Kong residents who underwent semen analysis (SA) as part of the fertility assessment at the andrology laboratory of Prince Wales Hospital, The Chinese University of Hong Kong, between April 2023 and August 2024, were recruited. Semen samples were collected from the participants who agreed to donate their leftover samples for research. An additional sperm test was performed only for subjects with sufficient specimens after clinical use.

Participants with medical conditions which may affect the semen quality were excluded. The medical conditions included: (1) Azoospermia; (2) Andrological conditions which are known to affect semen parameters including genetic conditions (such as AZFc microdeletion), history of mumps orchitis, severe varicocele, undescended testis, history of testicular torsion or scrotal injury, congenital bilateral absence of vas deferens (CBAVD) and urogenital infection; (3) Taking medication known to affect semen parameters, including steroid, finasteride, calcium channel blockers; (4) History of malignant disease; (5) Known mental disorders; (6) Drug abuse; (7) Refusal to donate semen samples.

## Semen Analysis

Routine semen analysis services are conducted in the Prince of Wales Hospital, Hong Kong, and the laboratory is accredited and joined an external quality assurance - External Quality Assessment Services (UK) program. In brief, the semen samples were obtained by masturbation after 2–7 days of sexual abstinence and semen analysis was performed manually according to World Health Organization guidelines (6th version) [80]. After liquefaction, semen samples were evaluated for motility, concentration, and morphology using the Nikon Eclipse E400 trinocular microscope. Improved Neubauer counting chambers were used for the sperm concentration at ×400 magnification. Sperm motility was classified into progressive motility (rapid and slow), non-progressive motility, and immotility. Sperm normal morphology was evaluated using the Diff-Quik method (RAL diagnostics, Ref. 720555-0000) at ×1,000 oil-immersion magnification. For samples with total motility less than 40%, additional sperm vitality assessments were conducted. Eosin-nigrosin stain was used to distinguish the viable and non-viable sperm.

## Total Mercury and Methylmercury Analyses

After semen characterization, remaining sample of whole semen from each patient was used for Hg analysis. In brief, each whole semen sample was vortexed for 30 s to ensure homogeneity, from which a portion of the well-mixed whole semen was used for subsequent THg and MeHg analysis. Another aliquot of the whole semen was transferred into a separate Eppendorf tube and centrifuged for 10 min at 3,000 g to obtain seminal plasma. The paired whole semen and seminal plasma samples were stored at -20 °C until THg and MeHg analysis.

Prior to THg analysis, whole semen and seminal plasma were thawed and vortexed for 30 s, after which 100 µL of each sample was pipetted into a pre-baked, Hg-free ceramic boat. THg concentrations were then measured by thermal decomposition of the sample follow by measurement with an atomic absorption spectroscopy (MA-3000, Nippon Instruments Corporation). A Hg standard solution (NIST SRM 3133, National Institute of Standards and Technology, Gaithersburg, Maryland, USA) was used to develop the calibration curves. A certified reference material (Seronorm™ Trace Elements Whole Blood L-3 RUO) was included along each batch of samples for quality assurance and quality control, and THg recoveries ranged from 93 to 120% (*n* = 35).

For MeHg analysis, 100 µL of whole semen or seminal plasma was digested with 4.6 M trace-metal grade nitric acid at 60 °C for 16 h [34]. After cooling, an aliquot of the acid digest was added to low-Hg deionized water with acetate buffer in a borosilicate glass vial, and the pH was adjusted to 4.9 with 4.6 M potassium hydroxide. Species of Hg in the mixture were ethylated by the addition of ice-cold 1 % sodium tetraethylborate and allowed to react on a shaking table for 30 min. MeHg was analyzed by Tekran 2700 automated MeHg analyzer (Tekran Instruments Corporation, Toronto, Ontario, Canada). The calibration curve was developed using a MeHg standard solution obtained from Brooks Rand Instrument (Seattle, Washington, USA). Quality control and assurance were performed using method blanks, sample replicates, and certified reference materials (Seronorm™ Trace Elements Whole Blood L-3 RUO). Recovery of MeHg from certified reference materials ranged from 83 to 116% (*n* = 29).

THg and MeHg measurements were conducted in duplicate, and the average value was reported. Any duplicate analysis with a relative standard deviation exceeding 20% was repeated but all duplicate analysis had that < 20%. The method detection limits for both THg and MeHg were established to be 0.01 µg/L (or 10 ng/L). Inorganic Hg (iHg) was calculated by subtracting the MeHg concentration from the THg concentration in each sample.

## Data Processing and Statistical Analysis

Statistical analyses were conducted using OriginPro 2024 (OriginLab Corporation, Northampton, Massachusetts, USA) and IBM SPSS^®^ Statistics version 31 (IBM Corp., Armonk, New York, USA). Distributions of semen quality parameters and Hg species concentrations were assessed and log-transformed when appropriate. Differences in Hg levels between semen quality groups (normal *vs.* poor) were compared using the Mann-Whitney U test. For comparisons across multiple categories defined by the number of abnormal semen parameters (0–6), group differences were assessed using the Kruskal-Wallis test, followed by Bonferroni-adjusted post hoc pairwise comparisons to account for multiple testing. To examine associations between Hg exposure and the burden of abnormal semen parameters, we fitted univariable ordinal logistic regression models with the number of abnormal semen parameters (ordered categories) as the outcome and ln-transformed Hg species concentrations as predictors. Spearman’s rank correlation coefficients were calculated to assess associations between Hg species concentrations and semen quality parameters.

Regression analyses were used to examine associations between Hg levels and semen quality parameters, with normal morphology and total motility as key outcomes. Normal morphology (%) and total motility (%) were both converted to a proportion and analyzed as logit(x) = ln[x ÷ (1-x)] in linear regression models. Because logit is undefined for x = 0, the proportion of 0 % was replaced with 0.1 %. For each Hg species (THg, MeHg, iHg), the amount of Hg per 10^6^ sperm cells (i.e., Hg per 10^6^ sperm) was calculated as Hg per ejaculate divided by total sperm count, where Hg per ejaculate (ng) = semen Hg concentration (ng/mL) × semen volume (mL). Predictors entered the models (Hg per 10⁶ sperm, THg per ejaculate, and total sperm count) were log_2_-transformed. β represents the change in logit (x) per doubling of the predictor. Three models were fitted: a crude model with Hg per 10^6^ sperm (Model 1), a primary model additionally adjusting for total sperm count, the denominator of the Hg per 10^6^ sperm metric (Model 2), and a decomposition model (Model 3) separating the numerator and denominator components of Hg per 10⁶ sperm to evaluate whether the observed association was more closely related to the Hg numerator (Hg per ejaculate) or the denominator (total sperm count). Percentage-point changes were estimated at the median normal morphology and motility.

## Results

### Semen Quality Parameters

The study included 250 participants with a median age of 36 years old (range: 26–63 years old). The median ejaculatory abstinence period was 3 days in both the normal and poor semen quality groups, and all semen samples liquefied within 30 min. There were no statistically significant differences between the normal and poor quality groups in terms of age (*p* = 0.1571) or abstinence days (*p* = 0.7881), indicating similar basic characteristics between groups (SI Table S1). Overall, 93 semen samples showed normal quality while 157 semen samples showed poor sperm quality, with the latter being defined as having at least one parameter below the reference limit (SI Table S2). Among the 157 samples showing poor sperm quality (62.8 % of total), the majority would have 1–3 abnormal parameters (combined for 52.8 % of total) with the remaining 10.0 % of total having 4–6 abnormal parameters (SI Table S3). Based on the reference value for human semen characteristics [80], we found that about half of patient samples were below reference limit for normal morphology, while for other semen quality parameters about 10–23 % of patient samples were below the reference limit of respective parameters (Table 2).

**Table 2:** Semen quality parameters in the study population

Semen quality parameters	Min	P25	Median	P75	Max	Patients below reference limit (%)
Concentration (10^6^/mL)	0.5	19.3	38.2	57.6	198.5	52 (20.8 %)
Total sperm count (10^6^/ejaculate)	1.1	42	87.5	158	474	58 (23.2 %)
Total motility (%)	0	49	54	58	70	31 (12.4 %)
Progressive motility (%)	0	45	50	54	64	24 (9.6 %)
Normal morphology (%)	0.5	1.8	3.4	5.1	10.1	127 (51.4 %)
Semen volume (mL)	0.08	1.7	2.65	3.7	7.4	38 (15.2 %)

## Mercury Levels

**Fig. 1:**
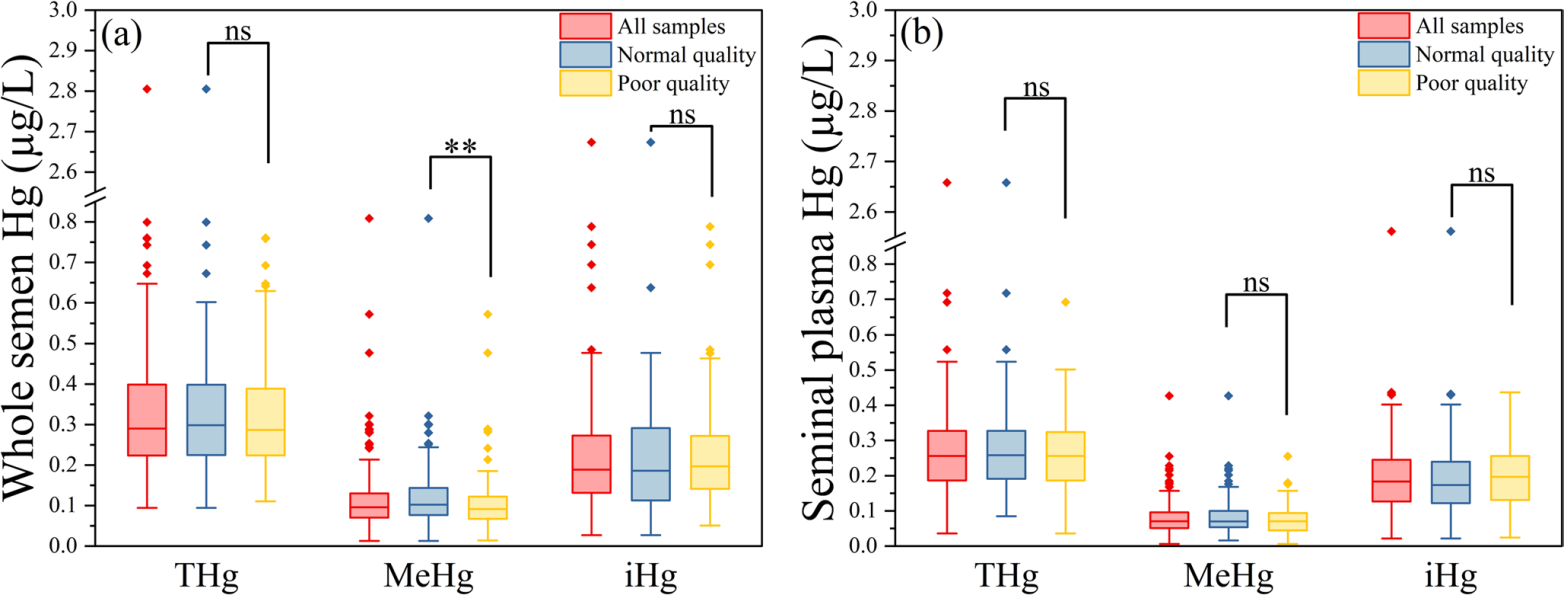
Distribution of mercury (Hg) species concentrations in (**a**) whole semen and (**b**) seminal plasma among all samples and by semen quality group. Data are shown as boxplots (median, interquartile range; whiskers = 1.5⋅ interquartile range). Pairwise comparisons between the normal and poor quality groups were performed using the Mann-Whitney U test. Only whole semen MeHg differed between normal and poor quality (*p =* 0.0072); all other comparisons were non-significant. ns, not significant, ^**^p < 0.01

Mean and standard deviation of THg in all 250 patient samples were 0.35 ± 0.23 µg/L in whole semen and 0.28 ± 0.19 µg/L in seminal plasma (SI Table S2), and thus roughly 80 % of THg in semen was found in the seminal plasma while the rest would be associated with sperm cells that we could calculate the Hg burden below. Among samples with normal vs. poor quality, even though the normal quality group had ~ 10 % higher THg than the respective poor quality group (i.e., semen: 0.36 ± 0.31 µg/L (normal) *vs.* 0.34 ± 0.17 µg/L (poor); seminal plasma: 0.30 ± 0.28 µg/L (normal) *vs.* 0.27 ± 0.10 µg/L (poor); SI Table S2), no statistical differences (*p* < 0.05) were observed among these different quality groups in both semen (Fig. 1a) and seminal plasma (Fig. 1b).

Regarding Hg speciation, semen had an average of ~ 65 % of THg as iHg and ~ 70 % of THg as iHg in seminal plasma. Thus, MeHg would be only for a smaller proportion for both semen (~ 35 %) and seminal plasma (~ 30 %). Again, we did not observe significant differences (*p* > 0.05) in semen iHg levels among samples of normal vs. poor quality groups (Fig. 1a). Interestingly, we identified that semen MeHg level was significantly higher in the normal quality group than poor quality group (*p* < 0.01) **(**Fig. 1a**)**, however, we did not observed such significant difference in seminal plasma (*p* > 0.05) even though the normal quality group had higher MeHg than the poor quality group in seminal plasma **(**Fig. 1b**)**.

**Fig. 2:**
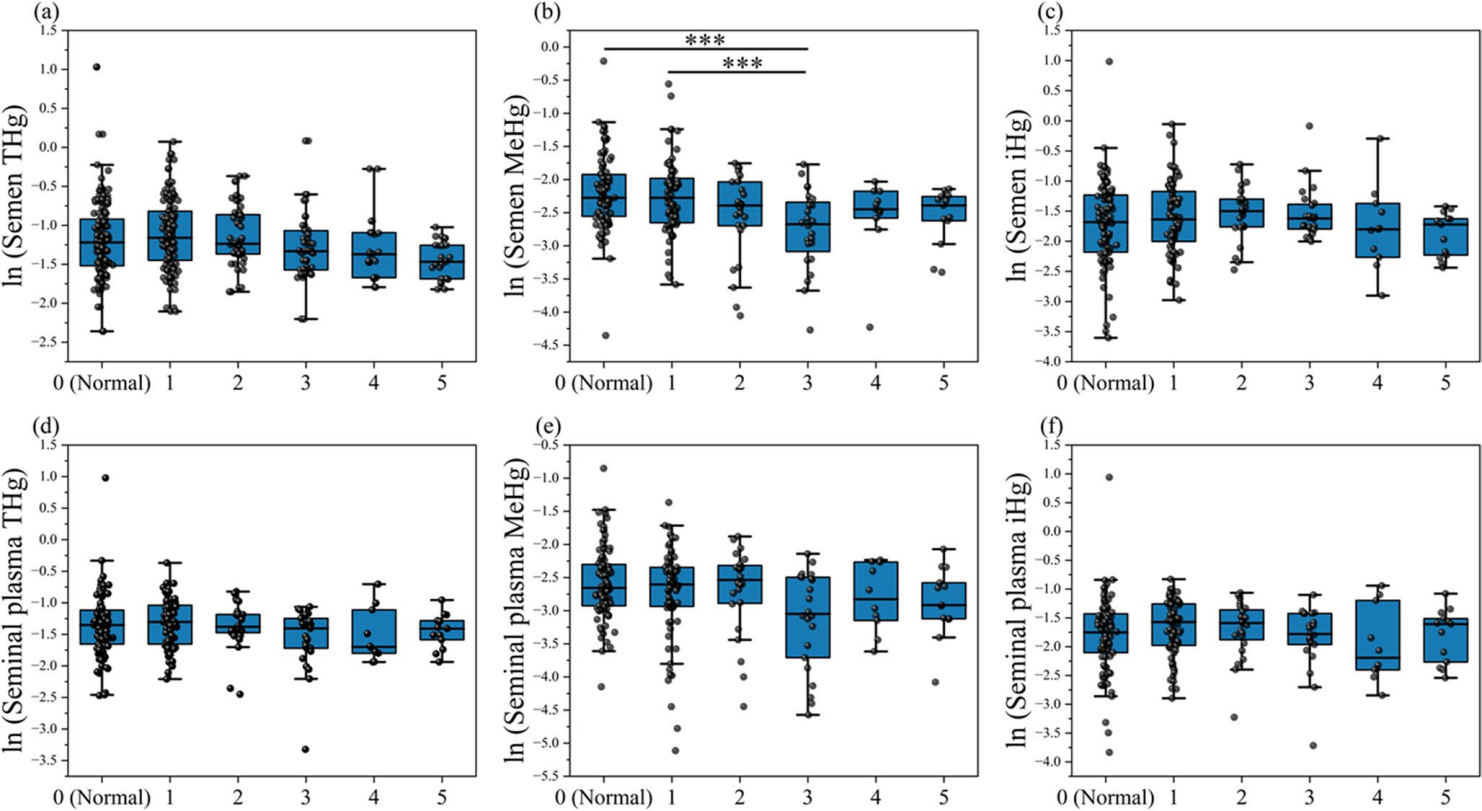
Distribution of ln-transformed Hg species in semen (**a**-**c**) and seminal plasma (**d**-**f**) by the number of abnormal semen parameters (0 = normal; 1–5 = increasing abnormalities). Data are shown as boxplots (median, IQR; whiskers = 1.5⋅ IQR). ^***^p < 0.001

Kruskal–Wallis tests were applied to assess whether Hg species concentrations differed across impairment categories defined by the number of abnormal semen quality parameters (higher counts indicate greater impairment). The category with six abnormal parameters was excluded due to the limited sample size (*n* = 1 or 0.4 % of total cases). As summarized in SI Table S4, only semen MeHg level showed a significant overall difference across categories (*p* = 0.001), whereas semen THg and iHg, as well as all three Hg species in seminal plasma, were not significant (all *p* > 0.05) (Fig. 2). Interestingly, Fig. 2b illustrated that samples with multiple abnormalities tended to have lower MeHg compared with those with no abnormal parameters (e.g., 0), while no clear separation was observed for THg (Fig. 2a) nor iHg (Fig. 2c).

**Fig. 3:**
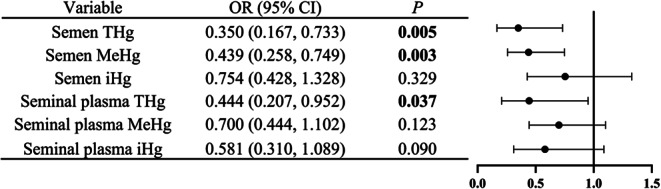
Ordinal logistic regression of ln-transformed Hg species levels predicting the number of abnormal semen parameters, adjusted for participant age and abstinence days. Separate models were fitted for semen and seminal plasma THg, MeHg, and iHg. Values are OR (95 % CI); Statistically significant values are identified in boldface

In univariable ordinal logistic regression models, after adjusting the participant age and abstinence days, higher semen THg and MeHg were associated with lower chances being in a more impaired category (THg: OR = 0.350, *p* = 0.005; MeHg: OR = 0.439, *p* = 0.003) (Fig. 3). Similarly, seminal plasma THg was inversely associated with impairment severity (OR = 0.444, *p* = 0.037), whereas both seminal plasma MeHg and seminal plasma iHg were not statistically significant (*p* = 0.123 and *p* = 0.090, respectively).

## Correlations Between Hg Species Levels and Sperm Quality Parameters

**Fig. 4:**
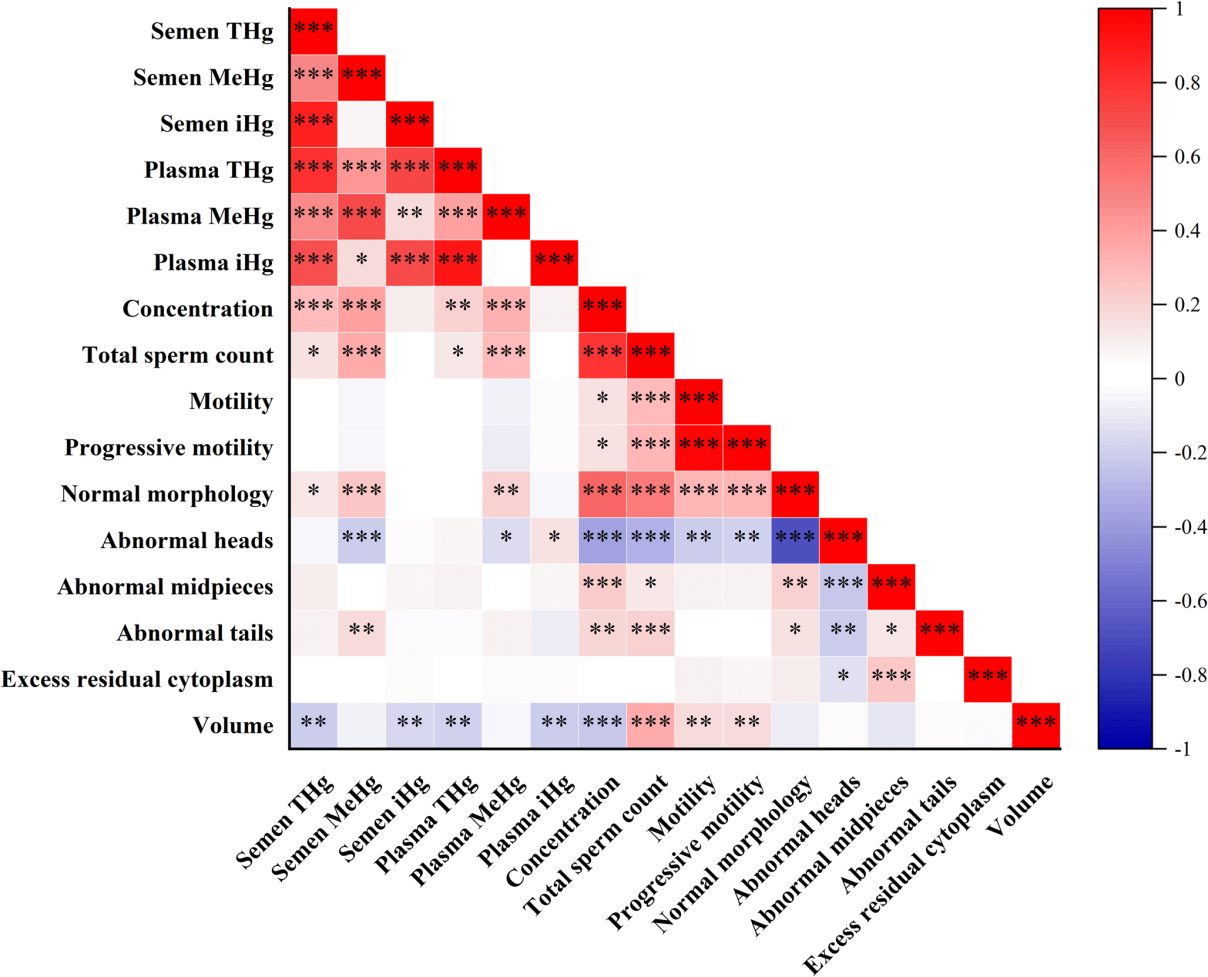
Correlations of mercury (Hg) species and semen parameters in all populations. The color bar reflects correlation strength and direction (-1 to 1), and asterisks indicate significance (^*^p < 0.05, ^**^p < 0.01, ^***^p < 0.001)

Among all test subjects, Hg species concentrations (i.e., THg, iHg, and MeHg) were positively correlated with each other in both semen and seminal plasma, and across the two matrices, indicating consistency between matrix specific Hg measures (Fig. 4). Specifically, semen concentrations of THg and MeHg were positively correlated with sperm concentration and total sperm count (e.g., semen THg: *r* = 0.29 with concentration and *r* = 0.16 with total sperm count; semen MeHg: *r* = 0.38 and *r* = 0.35, respectively), whereas correlations with total motility and progressive motility were not significant across Hg species. Semen and seminal plasma MeHg showed modest positive correlations with normal morphology (semen MeHg: *r* = 0.26; seminal plasma MeHg: *r* = 0.21) and inverse correlations with abnormal heads (semen MeHg: *r*=-0.21; seminal plasma MeHg: *r*=-0.16), while correlations with specific morphology subtypes were otherwise heterogeneous. Semen volume was inversely correlated with Hg species concentrations, most prominently for THg and iHg, consistent with dilution induced variation in Hg concentrations.

In contrast, the pattern differs remarkably when we normalize Hg species to fixed number of sperm cell. Specifically, different Hg species normalized to sperm cells (e.g., amount of Hg per 10^6^ sperm; log2-transformed) was inversely associated with normal morphology and total motility (both logit-transformed) in crude models (Table 3). For normal morphology, the crude model coefficients for THg, MeHg and iHg were − 0.335, -0.301, and − 0.283 (all *p* < 0.001), respectively, accounting for ~ 31 % to ~ 40 % of variance depending on Hg species. After adjusting for total sperm count (primary model), the negative associations remained statistically significant for all three Hg species but were somewhat attenuated (THg β = -0.218, *p* < 0.001; MeHg β = -0.117, *p* = 0.003; iHg β = -0.141, *p* < 0.001). Total sperm count showed a positive association with normal morphology in the primary model (THg β = 0.134; MeHg β = 0.232; iHg β = 0.193, all *p* < 0.001). Predictions indicated that, at the median normal morphology (3.4 %), a doubling of THg/MeHg/iHg normalized to sperm count corresponded to predicted normal morphology decreases of -0.94 pp, -0.86 pp, -0.82 pp, respectively, in crude models. After adjusting for total sperm count, the corresponding decreases became smaller: -0.65 pp, -0.36 pp, -0.43 pp (SI Table S5).

**Table 3:** Associations of mercury (Hg) species with sperm morphology and total motility using logit-transformed outcomes. Linear regression models were fitted for normal morphology (%) and total motility (%) with log2-transformed Hg species levels

Predictor	THg: β (95% CI)	*p*	MeHg: β (95% CI)	*p*	iHg: β (95% CI)	*p*
Model 1 (crude)						
Normal morphology (%)						
log2 (Hg per 10^6^ sperm)	-0.335 (-0.387, -0.283)	< 0.001	-0.301 (-0.358, -0.244)	< 0.001	-0.283 (-0.330, -0.235)	< 0.001
Model 2 (primary)						
log2 (Hg per 10^6^ sperm)	-0.218 (-0.303, -0.132)	< 0.001	-0.117 (-0.195, -0.040)	0.003	-0.141 (-0.214, -0.068)	< 0.001
log2 (total sperm count)	0.134 (0.055, 0.213)	< 0.001	0.232 (0.160, 0.304)	< 0.001	0.193 (0.115, 0.271)	< 0.001
Model 3 (decomposition)						
log2 (total Hg per ejaculate)	-0.218 (-0.303, -0.132)	< 0.001	-0.117 (-0.195, -0.040)	0.003	-0.141 (-0.214, -0.068)	< 0.001
log2 (total sperm count)	0.352 (0.300, 0.404)	< 0.001	0.350 (0.295, 0.405)	< 0.001	0.334 (0.285, 0.384)	< 0.001
Total motility (%)						
Model 1 (crude)						
log2 (Hg per 10^6^ sperm)	-0.179 (-0.219, -0.139)	< 0.001	-0.180 (-0.221, -0.139)	< 0.001	-0.148 (-0.185, -0.111)	< 0.001
Model 2 (primary)						
log2 (Hg per 10^6^ sperm)	-0.078 (-0.143, -0.012)	0.020	-0.080 (-0.137, -0.023)	0.006	-0.033 (-0.089, 0.023)	0.245
log2 (total sperm count)	0.115 (0.055, 0.174)	< 0.001	0.127 (0.075, 0.180)	< 0.001	0.154 (0.095, 0.213)	< 0.001
Model 3 (decomposition)						
log2 (total Hg per ejaculate)	-0.078 (-0.143, -0.012)	0.020	-0.080 (-0.137, -0.023)	0.006	-0.033 (-0.089, 0.023)	0.245
log2 (total sperm count)	0.192 (0.153, 0.232)	< 0.001	0.207 (0.166, 0.248)	< 0.001	0.187 (0.149, 0.225)	< 0.001

Model 1: crude model; Model 2: primary model adjusted for total sperm count; Model 3: decomposition model separating Hg per 10^6^ sperm into numerator (total Hg per ejaculate) and denominator (total sperm count). Normal morphology (%) and total motility (%) were analyzed as logit(x), where x = %/100. Predictors were log2-transformed; β represents the change in logit(x) per doubling of the predictor. N and R² (Models 1/2/3): normal morphology THg (*N* = 246; R² = 0.397/0.422/0.422), MeHg (*N* = 238; R² = 0.309/0.408/0.408), iHg (*N* = 237; R² = 0.369/0.425/0.425); total motility THg (*N* = 247; R² = 0.255/0.295/0.295), MeHg (*N* = 240; R² = 0.246/0.310/0.310), iHg (*N* = 238; R² = 0.219/0.295/0.295)

Results are presented as β (95% CI) with *p*-values for THg, MeHg and iHg. All models were adjusted for participant age and abstinence days

For total motility, crude models showed negative associations for all Hg species (THg β = -0.179; MeHg β = -0.180; iHg β = -0.148; all *p* < 0.001) **(**Table 3**)**. In primary models, the associations were reduced and became non-significant for iHg (THg β = -0.078, *p* = 0.020; MeHg β = -0.080, *p* = 0.006; iHg β = -0.033, *p* = 0.245). Median-referenced predictions at the median motility of 54 % showed that doubling THg/MeHg/iHg per 10⁶ sperm would decrease motility with − 4.47 pp, -4.49 pp, -3.69 pp in crude models. After adjustment in primary models, estimated decreases were − 1.94 pp, -1.99 pp for THg and MeHg (SI Table S6). In decomposition models, replacing Hg normalized to sperm count with its numerator (i.e., total Hg per ejaculate) and denominator (i.e., total sperm count) yielded similar negative coefficients for total Hg per ejaculate as in the primary models, while total sperm count retained positive associations with both outcomes **(**Table 3**)**.

## Discussion

### Unexpectedly High Proportion of Inorganic Hg in Whole Semen and Seminal Plasma

In our study population, iHg accounted for an average of 65 % of THg in whole semen and 70 % in seminal plasma, which is a pattern that differs from those commonly reported for other human biomarkers. For example, iHg often constitutes a smaller fraction of THg in human hair (iHg% ≈ 10–20 %) and whole blood (average iHg% ≈ 25 %) [8, 76], whereas urinary THg is largely dominated by iHg (> 98 %) due to renal elimination of inorganic Hg species and metabolic demethylation of MeHg in liver and kidney [20, 36, 67]. To our knowledge, previous clinical studies have reported only THg concentrations in semen matrices (Table 1), while different Hg species have not been measured [4, 18, 26, 41, 77]. Therefore, our current study represents the first attempt to do both THg and MeHg measurements on semen samples, and our demonstration of high iHg fraction in these samples is important. However, the determinants and mechanisms underlying the surprisingly high iHg fraction in semen and seminal plasma are not known at the moment. To evaluate whether this result could simply reflect unusually high background iHg/Hg⁰ exposure in Hong Kong, we compared urinary Hg levels across reference populations (SI Table S7). Urinary Hg, a commonly used biomarker of iHg/Hg⁰ exposure [46, 67], was found to be comparable between Hong Kong and other general populations [16, 58, 65, 82], but much lower than those reported in the Hg contaminated areas such as Wanshan mining area in Chinese mainland [46] as well as gold miners in Zimbabwe [51] and Mexico [23]. These comparisons imply that the high iHg fraction observed in whole semen and seminal plasma should not be attributed to elevated background iHg exposure in Hong Kong. Based on the current knowledge of Hg toxicokinetics, we proposed two plausible mechanisms.

First, iHg measured in semen can reflect contributions from multiple upstream sources, including systemic iHg following direct exposure to inorganic Hg compounds, iHg generated by oxidation of element Hg after inhalation exposure, and iHg formed via in vivo demethylation of MeHg. Salts of Hg (iHg) are used as an additive in prescription drugs (e.g., laxatives) and products including cosmetics and teeth powdering [52]. After absorption, iHg formed conjugates with sulfhydryl-containing molecules (e.g., amino acids, peptides), which is important in its internal transport and distribution [9]. iHg accumulate primarily in the kidney as well as the intracellular accumulation in the interstitial Leydig cells and the Sertoli cells of the seminiferous tubules [27]. Following inhalation of Hg^0^ through occupational activities and environmental sources, it is oxidized rapidly to iHg by catalase in the blood and distributed to multiple tissues, including testicle [25, 37, 46]. Consequently, iHg may enter reproductive fluids through secretions from reproductive tissues that contribute to semen iHg accumulation. Alternatively, semen iHg may also partly originate from in-vivo demethylation of dietary MeHg. For example, demethylation by anaerobic gut microbiota has been proposed as an intermediate step in MeHg elimination [14], and additional demethylation pathways have been described in liver and kidney [44, 45]. The resulting iHg is primarily eliminated via feces and urine [61, 67]. Even though direct evidence for reproductive organ demethylation is lacking, as mentioned above, reproductive system can be a site of iHg deposition, and any demethylation occurring before or within reproductive tissues could yield iHg that is then retained and enters semen. Overall, semen iHg% may reflect a shared iHg pool by iHg exposure history and in vivo metabolic conversion of Hg^0^ and MeHg, with subsequent transfer into reproductive fluids. Pairing semen Hg species levels with other biomarkers (e.g., urinary Hg for iHg, hair Hg for MeHg) may help clarify the dominant contributors.

Second, we demonstrated that iHg% was even higher in seminal plasma (70 %) than in whole semen (65 %), our results suggest that iHg is relatively more concentrated in the seminal plasma fraction than in the cellular fraction (e.g., spermatozoa). This shifts attention to the seminal vesicles and prostate, the primary sources of seminal plasma. We use kidney and urine as a reference because the kidney is a well characterized epithelium system for iHg disposition, and urinary Hg is typically dominated by iHg due to transporter mediated handling of mercuric conjugates [78]. Moreover, both renal tubules and accessory sex glands move selected solutes from the circulation into the luminal fluid (urine and glandular secretions), making the renal pathway a useful conceptual analogy [50]. Therefore, the second explanation is that secretory epithelia of seminal vesicles and prostate may handle and accumulate iHg in a way conceptually analogous to the kidney. In renal proximal tubules, iHg or Hg^2+^ is transported predominantly as thiol conjugates, with SLC family uptake transporters (e.g., amino acid transporters, organic anion transporters) mediating cellular entry, while ABC efflux transporters (e.g., MRP2 and BCRP) can export mercuric conjugates into tubular fluid for urinary excretion [10]. Notably, a study of transporter expression and localization in the human seminal vesicle also showed that the seminal vesicle epithelium expressed a broad panel of SLC and ABC transporters and exhibited luminal localization of BCRP [50], supporting an active secretory function that could potentially facilitate transfer of Hg^2+^-thiol conjugates into seminal plasma.

### Inverse Association Between Hg and Semen Impairment

Using the number of abnormal semen parameters as an ordinal indicator of impairment severity [33], we observed inverse associations between concentration-based Hg measures (µg/L) and impairment severity (Fig. 3). Instead of suggesting a protective effect of Hg, this counterintuitive pattern is plausibly consistent with high seafood consumption in Hong Kong [83]. Specifically, the average fish consumption per capita in Hong Kong is 65.5 kg per year, which is at least three times higher than the global average of 19.7 kg per capita per year [17]. Consequently, Hong Kong residents are likely to have higher dietary MeHg exposure. Blood THg level, as an acceptable biomarker for MeHg exposure in most communities [8] was substantially higher in Hong Kong residents (6.08 µg/L; [70]) than in the group of Non-Hispanic Asian in the United States (1.24 µg/L; [58]). In contrast, urine THg level, as a commonly used biomarker of iHg/Hg^0^ exposure, was similar among residents of Hong Kong (0.55 µg/g creatinine; [82]) and the United States (0.58 µg/g creatinine; [58]). Taken together, these patterns suggest that Hg exposure in our cohort should be strongly influenced by fish-derived MeHg than by iHg sources. Consistent with this interpretation, local studies have reported measurable MeHg level (median 45 ng/g wet wt.) in commonly consumed fish species from markets in Hong Kong [74].

Fish intake, however, co-delivers nutrients that have been linked to improved semen quality, particularly ω-3 fatty acids (e.g., EPA and DHA) [63, 64]. Afeiche et al., [2] found that higher fish intake was significantly associated with higher total sperm count and normal morphology. In Hong Kong, Chung et al., [19] further showed that 25 of the 88 commonly consumed fish species had both MeHg < 0.1 µg/g wet wt. and EPA + DHA > 3 mg/g wet wt., suggesting that some commonly consumed fish should provide favorable amount of ω-3 fatty acids with relatively low MeHg exposure. Collectively, these findings support the interpretation that, in a high fish and seafood consuming population such as Hong Kong residents, semen MeHg concentration may function as a dietary marker of fish intake, capturing both MeHg exposure and the accompanying nutritional benefits. Therefore, the observed inverse association between Hg levels and semen impairment severity can be better explained by the overall dietary pattern (MeHg exposure occurring alongside beneficial nutrients) rather than by a beneficial effect of Hg itself. Nonetheless, this pattern may not be considered to be generalized because environmental Hg exposure and dietary structures vary substantially according to local culture and geographic location.

### Different Testicular Access of MeHg

Beyond the dietary explanation, another reason why semen MeHg, but not iHg, was significantly correlated with total sperm count (Fig. 4) might be different testicular access. MeHg can form thiol conjugates (e.g., MeHg-L-cysteine) that mimic the neutral amino acid methionine and be transported by the L-type large neutral amino acid transporter LAT1 [81], which is also highly expressed in the testis [60]. If LAT1-mediated transport increases MeHg delivery into testicular compartments behind the blood-testis barrier, MeHg may be incorporated into developing germ cells and carried by spermatozoa [43]. This could contribute to the observed positive correlation between semen MeHg and total sperm count (Fig. 4), without implying a beneficial effect of MeHg on spermatogenesis.

### Comparison with Previous Studies

Our findings indicate that semen THg and semen MeHg levels are significantly associated with sperm cell concentration, total sperm count and sperm normal morphology (Fig. 4). Moreover, higher semen THg and semen MeHg levels were significantly associated with lower odds of severe semen impairment after adjusting for potential confounders (Fig. 3). These results are consistent with previous human studies reporting positive associations between Hg biomarker levels and semen quality or in vitro fertilization outcomes [41, 56]. A possible explanation, also mentioned in previous studies, is that these positive associations reflect higher consumption of seafood intake, which may in fact benefit semen quality.

For studies reporting non-significant or negative associations between Hg biomarkers and semen quality [4, 18, 24, 35, 54, 55], we found that several explanations are plausible. First, difference in dietary patterns and dominant Hg exposure sources may modify the observed associations. Individuals exposed to Hg other than fish consumption may not experience the same sperm benefit as our cohort in Hong Kong. Second, biological matrices differ in their relevance to male reproductive toxicity. Whole semen and seminal plasma can directly reflect toxic effects on the reproductive system, whereas blood and hair mainly reflect overall Hg exposure. Third, these prior studies assessed only THg. For example, in our cohort, seminal plasma MeHg was significantly correlated with sperm normal morphology, while seminal plasma THg was not (Fig. 4), indicating that studies measuring only THg may fail to detect this association. Fourth, differences in study populations may also contribute to the inconsistent findings, as participants recruited from the general population, occupational settings, or infertility clinics may differ in baseline reproductive health and susceptibility to Hg exposure. Fifth, variability in exposure levels and exposure ranges across studies may influence the observed associations, because a detectable relationship may only emerge within certain exposure ranges or may differ between low and high exposure populations.

### Mercury Burden in Sperm

To improve interpretability at a sperm cell level, Hg normalized to sperm count (i.e., 10⁶ sperm cells) was used to interpret the bioaccumulation data. Accordingly, we used three complementary models: Model 1 (*crude*) quantified the overall association between Hg normalized to 10⁶ sperm and outcomes. However, because Hg per 10⁶ sperm is a ratio metric that includes total sperm count in its denominator, its variability can be strongly coupled to the denominator and cause misleading inference. Therefore, Model 2 (*primary*) additionally adjusted for total sperm count to evaluate whether at a given sperm count, Hg per 10⁶ sperm is still linked to normal morphology and motility. Model 3 (*decomposition*) decomposed the ratio into its numerator (total Hg per ejaculate) and denominator (total sperm count) to clarify which component that can drive the association. Across Hg species, higher Hg per 10⁶ sperm was consistently associated with lower normal morphology in crude models and remained inversely associated after total sperm count adjustment (attenuated but robust), while decomposition results showed that the negative signal tracked the numerator (total Hg per ejaculate) alongside a strong, independent positive association of total sperm count with morphology. For total motility, crude inverse associations were observed across species, but after adjusting for total sperm count, the association persisted for THg and MeHg but was no longer significant for iHg, pointing to the potential form-specific differences in disrupting sperm motility.

Using model 2 for THg, a doubling of THg normalized to sperm count (10⁶ sperm) corresponded to a decrease of -0.65 pp in normal morphology (SI Table S5) and a decrease of -1.94 pp in total motility (SI Table S6). When these predicted shifts were applied to our cohort, the prevalence below normal morphology reference limit substantially increased from 51.4 to 69.6 %. In contrast, the prevalence below the total motility reference limit changed merely from 12.4 to 14.0 %. Taken together, these results support that Hg normalized to sperm cell count (i.e., 10⁶ sperm) can be a robust proxy of sperm Hg burden, and that the negative signal is primarily linked to total Hg burden per ejaculate rather than changes dominated by total sperm count.

To interpret these findings, we examined experimental evidence regarding the biological effects of Hg on sperm. Boujbiha et al., [11] and Boujbiha et al., [12] demonstrated that iHg affect sperm cells primarily by inducing oxidative stress within the rat testis. Subchronic HgCl₂ exposure reduced epididymal sperm count and motility, caused degeneration of seminiferous tubules, and led to a decline in male reproductive performance, implying impairment of both sperm quality and functional fertility. The observed increase in testicular lipid peroxidation, together with suppression of antioxidant enzymes, supports the view that redox imbalance is a key mediator of iHg induced sperm damage. Mechanistically, iHg binds thiol (-SH) or seleno (-SeH) groups, depletes antioxidant defenses (e.g., glutathione-dependent systems), and promote reactive oxygen species (ROS) generation, thereby damaging sperm membranes, mitochondria and proteins needed for normal structure and movement [15, 43].

Evidence from Fossato da Silva et al., [29] indicates that MeHg can adversely affect sperm function in rats following only 14 days of exposure at doses comparable to Hg exposure from fish consumed by humans. MeHg consistently reduced progressive sperm motility, increased the proportion of non-progressive and immotile sperm, decreased sperm counts in the caput/corpus epididymis, accelerated epididymal transit, and increased sperm head abnormalities. These findings suggest that MeHg interferes not only with sperm quality but also with epididymal maturation and storage, both of which are critical for the development of fertilizing capacity. At the highest dosage, MeHg also reduced serum testosterone and epididymal androgen receptor immunoreactivity, supporting the notion that endocrine disruption by MeHg also contribute to reproductive toxicity [43].

Although sperm morphology has long been included in routine semen characterization, its predictive value for reproductive outcomes remains controversial. In a study of 501 couples who discontinued contraceptive use, a higher percentage of abnormal morphology was associated with a longer time-to-pregnancy [13]. However, another study showed that men with 0% normal forms still exhibited high rates of success without the need of assisted reproduction. The authors summarized that strict morphology alone should not be used to predict fertilization, pregnancy, or live birth potential [42]. Overall, current available evidence suggests sperm morphology has low predictive power for pregnancy success, for both natural and assisted reproduction [22, 31]. Biologically, motility is essential for sperm movement and fertilization, but it was not found to be an important factor associated with the probability of couples who discontinued contraception for 6 month and 12 months [73]. Together, our modeling results and the available evidence indicate that Hg burden per sperm negatively affects sperm quality, but the clinical implications for motility and morphology for fertility outcomes should be interpreted with caution.

### Summary, Limitations, and Future Studies

We investigated the associations between Hg species exposure and semen quality parameters in a cohort of 250 male subjects in Hong Kong. By measuring Hg species levels in paired whole semen and seminal plasma, we show that interpretation depends strongly on the Hg exposure metric. Volume-based Hg concentration measures (µg/L) may partly reflect seafood intake and correlated nutritional profiles and thus can exhibit inverse associations with impairment severity in this setting, which have been observed in a few previous studies (Table 1). In contrast, when Hg is normalized to sperm count (Hg per 10⁶ sperm) or likely sperm mass, higher Hg value was consistently associated with lower normal morphology and lower motility, suggesting a potential adverse association at a sperm cellular level.

We also observed a high iHg fraction in semen matrices, but determinants of this pattern remain unclear and warrant further investigation. Future studies should integrate more human biomarkers, more detailed questionnaire, and longitudinal repeated samples to clarify Hg sources, reduce residual confounding and confirm observed associations.

To the best of our knowledge, this is the first study to report Hg species concentrations (THg, MeHg, iHg) in paired semen matrices (whole semen and seminal plasma). Our work showed that analyzing different Hg species is important because MeHg and iHg differ in access to the male reproductive tract and in the pathways relevant to sperm function, therefore, THg alone may not capture differences in Hg species composition relevant to semen quality. Nonetheless, while gold mining does not exist in Hong Kong, we acknowledge that our study lacks detailed demographic, clinical, and lifestyle information (e.g., BMI, antibiotic use, dental amalgam, alcohol consumption, environmental and occupational exposure history, clinical records), raising the possibility of residual confounding. The dietary information was not collected at the time of semen sample collection. Therefore, discussion about the co-delivers of Hg and nutrients from fish consumption was based on relevant previous studies and statistical models could not be adjusted for fish intake. In addition, semen parameters and Hg measurements were based on a single snapshot from the individuals, which may not reflect their long-term Hg exposures. Finally, we recognized that because participants were recruited from an andrology laboratory for routine semen analysis, our results might not fully reflect the semen quality or Hg exposure patterns of the general male population in Hong Kong.

Future studies should combine longitudinal repeated reproductive matrices with other human biomarkers, such as hair Hg, urinary Hg, to better distinguish MeHg and iHg exposure pathways and to clarify how Hg is transferred into semen. In addition, hair samples can provide a timeline of Hg exposure, as each 1 cm hair segment from the scalp approximately represents one month of prior exposure [69]. By analyzing hair segments, we may estimate Hg exposure over the periods of spermatogenesis and sperm maturation. Furthermore, collected hair and urine samples can be used for Hg stable isotope analysis to explore whether integrated Hg sources differ across men with different semen quality. Numerous factors may also affect the observed associations between Hg and semen quality parameters, including occupations, dietary habits, fish consumption, dental amalgam, environmental/occupational exposure history, overall health, body mass index, and co-exposure to other substances like alcohol, traditional medicines, industrial chemicals, insecticides, and pesticides. Failure to account for these factors may attenuate or exaggerate the associations identified in the present study. Therefore, future studies should incorporate detailed questionnaire and clinical data to reduce residual confounding.

## Supplementary Information

Below is the link to the electronic supplementary material.

Supplementary Material 1 (DOCX 2.27 MB)

## Data Availability

No datasets were generated or analysed during the current study.
