# Supplementary material for: Sperm-Associated Mercury Species Levels Determine Its Impairment: Evidence from Paired Analyses of Whole Semen and Seminal Plasma
Source: Biol Trace Elem Res. 2026 Jun 16;204(9):7186–200. doi: 10.1007/s12011-026-05181-8 (PMC13437602; doi:10.1007/s12011-026-05181-8)
Supplement: Supplementary file 1 [file 12011_2026_5181_MOESM1_ESM.docx]

**Supplemental Information (SI)**

**Table S1**. Participant characteristics of the study population, stratified by semen quality (normal v.s. poor quality). Age and abstinence days are presented as median (range).

|  | N | Age | Abstinence days | Liquefaction time |
| --- | --- | --- | --- | --- |
| All participants | 250 | 36 (26-63) | 3 (1-15) | all < 30 mins |
| Normal quality participants | 93 | 36 (26-51) | 3 (1-7) | all < 30 mins |
| Poor quality participants | 157 | 37 (30-63) | 3 (2-15) | all < 30 mins |

**Table S2** Concentrations of Hg species (μg/L) in semen and seminal plasma by semen quality. Values are presented as mean (standard deviation).

|  |  | Semen | | | | Seminal plasma | | | |
| --- | --- | --- | --- | --- | --- | --- | --- | --- | --- |
|  | N | THg | MeHg | iHg | THg | | MeHg | iHg |  |
| All samples | 250 | 0.35 (0.23) | 0.11 (0.08) | 0.23 (0.21) | 0.28 (0.19) | | 0.08 (0.05) | 0.20 (0.18) |  |
| Normal quality | 93 | 0.36 (0.31) | 0.13 (0.10) | 0.24 (0.28) | 0.30 (0.28) | | 0.09 (0.06) | 0.21 (0.27) |  |
| Poor quality | 157 | 0.34 (0.17) | 0.10 (0.07) | 0.23 (0.15) | 0.27 (0.10) | | 0.07 (0.04) | 0.20 (0.09) |  |

**Table S3** Hg species levels (μg/L) in semen and seminal plasma by the number of abnormal semen parameters.

| Number of abnormal parameters | Number of samples | Semen | | | Seminal plasma | | |
| --- | --- | --- | --- | --- | --- | --- | --- |
|  |  | THg | MeHg | iHg | THg | MeHg | iHg |
| 0 | 93 (37.2%) | 0.36 | 0.13 | 0.24 | 0.30 | 0.09 | 0.21 |
| 1 | 76 (30.4%) | 0.36 | 0.12 | 0.24 | 0.28 | 0.08 | 0.21 |
| 2 | 29 (11.6%) | 0.34 | 0.09 | 0.23 | 0.27 | 0.08 | 0.20 |
| 3 | 27 (10.8%) | 0.31 | 0.07 | 0.24 | 0.23 | 0.05 | 0.18 |
| 4 | 11 (4.4%) | 0.30 | 0.09 | 0.22 | 0.24 | 0.07 | 0.17 |
| 5 | 13 (5.2%) | 0.24 | 0.08 | 0.16 | 0.24 | 0.06 | 0.18 |
| 6 | 1 (0.4%) | 0.33 | 0.11 | 0.22 | 0.38 | 0.04 | 0.34 |

**Table S4** Kruskal–Wallis test results for differences in mercury species concentrations across groups defined by the number of abnormal semen parameters in semen and seminal plasma.

| Matrix | Hg species | Kruskal–Wallis H | df | p value |
| --- | --- | --- | --- | --- |
| Semen | THg | 9.706 | 5 | 0.084 |
|  | MeHg | 20.015 | 5 | 0.001 |
|  | iHg | 5.552 | 5 | 0.352 |
| Seminal plasma | THg | 6.658 | 5 | 0.247 |
|  | MeHg | 10.189 | 5 | 0.070 |
|  | iHg | 5.264 | 5 | 0.385 |

**Table S5** Predicted normal morphology after a doubling of Hg exposure (median-referenced). Coefficients β (95% CI) from logit-transformed normal morphology models are presented for THg, MeHg and iHg. Predictions were calculated at the median normal morphology. Model 1: crude model; Model 2: primary model adjusted for total sperm count.

| Species | Predictor | β (95% CI) | Predicted morphology after doubling (%) | Δpp (95% CI)^1^ |
| --- | --- | --- | --- | --- |
| Model 1 (crude) |  |  |  |  |
| THg | Hg per 10^6^ sperm | -0.335 (-0.387, -0.283) | 2.46 | -0.94 (-1.07, -0.82) |
| MeHg | Hg per 10^6^ sperm | -0.301 (-0.358, -0.244) | 2.54 | -0.86 (-1.00, -0.71) |
| iHg | Hg per 10^6^ sperm | -0.283 (-0.330, -0.235) | 2.58 | -0.82 (-0.93, -0.69) |
| Model 2 (primary) |  |  |  |  |
| THg | Hg per 10^6^ sperm | -0.218 (-0.303, -0.132) | 2.75 | -0.65 (-0.87, -0.41) |
| MeHg | Hg per 10^6^ sperm | -0.117 (-0.195, -0.040) | 3.04 | -0.36 (-0.59, -0.13) |
| iHg | Hg per 10^6^ sperm | -0.141 (-0.214, -0.068) | 2.97 | -0.43 (-0.64, -0.22) |

^1^Δpp indicates the absolute change in percentage points, calculated as the predicted percentage after a doubling of Hg per 10^6^ sperm minus the reference median percentage of normal morphology (3.4%).

**Table S6** Predicted total motility after a doubling of Hg exposure (median-referenced). Coefficients β (95% CI) from logit-transformed total motility models are presented for THg, MeHg and iHg. Predictions were calculated at the median total motility. Model 1: crude model; Model 2: primary model adjusted for total sperm count.

| Species | Predictor | β (95% CI) | Predicted motility after doubling (%) | Δpp (95% CI)^1^ |
| --- | --- | --- | --- | --- |
| Model 1 (crude) |  |  |  |  |
| THg | Hg per 10^6^ sperm | -0.179 (-0.219, -0.139) | 49.53% | -4.47 (-5.47, -3.47) |
| MeHg | Hg per 10^6^ sperm | -0.180 (-0.221, -0.139) | 49.51% | -4.49 (-5.52, -3.47) |
| iHg | Hg per 10^6^ sperm | -0.148 (-0.185, -0.111) | 50.31% | -3.69 (-4.62, -2.77) |
| Model 2 (primary) |  |  |  |  |
| THg | Hg per 10^6^ sperm | -0.078 (-0.143, -0.012) | 52.06% | -1.94 (-3.57, -0.30) |
| MeHg | Hg per 10^6^ sperm | -0.080 (-0.137, -0.023) | 52.01% | -1.99 (-3.42, -0.57) |
| iHg^2^ | Hg per 10^6^ sperm | -0.033 (-0.089, 0.023) | 53.18% | -0.82 (-2.22, 0.57) |

^1^Δpp indicates the absolute change in percentage points, calculated as the predicted percentage after a doubling of Hg per 10^6^ sperm minus the reference median percentage of total motility (54%).

^2^Insignificant model.

**Table S7** Urinary mercury levels (μg/g creatinine) reported in Hong Kong and reference populations as indicators of background iHg/Hg^0^ exposure.

| Country/Region | Population | N | Urine Hg level  (μg/g creatinine) | Reference |
| --- | --- | --- | --- | --- |
| Hong Kong | Adolescents | 1939 | 0.55 | Yu et al., 2025 |
| United States | Non-hispanic Asian | 355 | 0.58 | CDC, 2024 |
| Spain | Adults | 1704 | 0.80 | Castaño et al., 2019 |
| Czech Republic | Adults | 305 | 0.40^1^ | Sharma et al., 2025 |
| Wanshan Hg mining area, China | Local residents | 160 | 8.32 | Li et al., 2015 |
| Zimbabwe | ASGM Miners^2^ | 207 | 3.98 | Mambrey et al., 2020 |
| Mexico | ASGM Miners^2^ | 34 | 503.4 | Díaz de León-Martínez et al., 2022 |

^1^Specific gravity-adjusted concentrations (μg/l)

^2^Artisanal and small-scale gold mining (ASGM)

**References**

Castaño, A., Pedraza-Díaz, S., Cañas, A.I., Pérez-Gómez, B., Ramos, J.J., Bartolomé, M., Pärt, P., Soto, E.P., Motas, M., Navarro, C., Calvo, E., Esteban, M., 2019. Mercury levels in blood, urine and hair in a nation-wide sample of Spanish adults. Science of The Total Environment 670, 262–270. <https://doi.org/10.1016/j.scitotenv.2019.03.174>

Díaz de León-Martínez, L., Ortega-Romero, M., Gavilán-García, A., Barbier, O.C., Carrizalez-Yáñez, L., Van-Brusel, E., Díaz-Barriga, F., Flores-Ramírez, R., 2022. Assessment of biomarkers of early kidney damage and exposure to pollutants in artisanal mercury mining workers from Mexico. Environ Sci Pollut Res 29, 13333–13343. https://doi.org/10.1007/s11356-021-16628-x

Li, P., Du, B., Chan, H.M., Feng, X., 2015. Human inorganic mercury exposure, renal effects and possible pathways in Wanshan mercury mining area, China. Environmental Research 140, 198–204. <https://doi.org/10.1016/j.envres.2015.03.033>

Mambrey, V., Rakete, S., Tobollik, M., Shoko, D., Moyo, D., Schutzmeier, P., Steckling-Muschack, N., Muteti-Fana, S., Bose-O’Reilly, S., 2020. Artisanal and small-scale gold mining: A cross-sectional assessment of occupational mercury exposure and exposure risk factors in Kadoma and Shurugwi, Zimbabwe. Environmental Research 184, 109379. <https://doi.org/10.1016/j.envres.2020.109379>

National Center for Environmental Health. National Report on Human Exposure to Environmental Chemicals. U.S. Department of Health and Human Services, Centers for Disease Control and Prevention. 2022. Updated December 2024. Accessed [Jan 20, 2026]. <https://dx.doi.org/10.15620/cdc:133100>

Sharma, B.M., Komprdová, K., Lörinczová, K., Kuta, J., Přibylová, P., Scheringer, M., Šebejová, L., Piler, P., Zvonař, M., Klánová, J., 2025. Human biomonitoring of essential and toxic trace elements (heavy metals and metalloids) in urine of children, teenagers, and young adults from a Central European Cohort in the Czech Republic. J Expo Sci Environ Epidemiol 35, 730–745. <https://doi.org/10.1038/s41370-024-00724-4>

Yu, J., Yang, A., Chow, E., Shi, M., Xue, Y., Tam, C.H., Chu, N.H., Cheung, S.H., Lau, E.S., Wu, H., Lui, J.N., Ma, R.C., Kong, A.P., Luk, A.O., Chan, Jones CM, Chan, I.H., Chan, M.H., Chan, Juliana CN, 2025. Environmental exposure to toxic and essential metals and glucose homeostasis in adolescents: A cross-sectional analysis of the Hong Kong School Children Cohort. Ecotoxicology and Environmental Safety 305, 119179. <https://doi.org/10.1016/j.ecoenv.2025.119179>
